# Efficient Up-Conversion ZnO Co-Doped (Er, Yb) Nanopowders Synthesized via the Sol-Gel Process for Photovoltaic Applications

**DOI:** 10.3390/ma15217828

**Published:** 2022-11-06

**Authors:** Marwa Derouiche, Rached Salhi, Samir Baklouti

**Affiliations:** 1 Laboratory of Advanced Materials, National Engineering School of Sfax, University of Sfax, Sfax 3038, Tunisia; 2 Laboratory of Electrochemistry and Physicochemistry of Materials and Interfaces, University of Grenoble Alpes-Savoie Mont Blanc, Grenoble INP, 38000 Grenoble, France

**Keywords:** sol-gel processes, up-conversion luminescence, ZnO, rare earth materials

## Abstract

In this study, undoped and (Erbium, Ytterbium) co-doped ZnO nanopowders were prepared using the sol-gel method and the supercritical drying of ethyl alcohol. Doping ZnO nanopowders were elaborated with 5 mol% of Er (5 Er: ZnO), 5 mol% of Er and 5 mol% of Yb (5 Er, 5 Yb: ZnO), and 5 mol% of Er and 10 mol% of Yb (5 Er, 10 Yb: ZnO) concentrations. The effects of the Yb concentration on the structural, morphological, photoluminescent, and electrical properties of the ZnO nanopowders were investigated. The main findings of this work were the crystallinization of all of the nanopowders in a hexagonal Wurtzite structure with a spheroidal morphology and a size of 60 nm. Hence, the doping concentration would affect the crystallinity and the morphology of the ZnO nanopowder. The UC (Up-Conversion) emissions were investigated under a 980 nm excitation. It was observed that (Er, Yb: ZnO) exhibited green, ranging between 525 nm and 550 nm and red up-converted emissions of 655 nm, due to the efficient energy transfer process between Er^3+^ and Yb^3+^. The absolute quantum yield percentage (QY %) of the doped nanopowders was measured as a function of power density at each up-converted emission. This would prove that (5 Er, 5 Yb: ZnO) had the highest QY percentage value of 6.31 ± 0.2% at a power density of 15.7 W/cm^2^. Additionally, it had the highest excited state lifetime for green and red emissions. Moreover, the Hall effect measures showed that the resistivity decreased while the electron mobility increased after doping, suggesting that most of rare earth ions were located in the interstitial positions. The carrier concentration increased after doping until (5 Er, 5 Yb: ZnO), suggesting that the Zn^2+^ ions substituted the RE^3+^ ions. Then, the carrier concentration decreased, suggesting that doping with higher concentrations would cause grain boundary defects. These findings would suggest that (5 Er, 5 Yb: ZnO) would have the best electrical properties and the lowest band gap energy (3.24 eV). Therefore, the presented preparation of the (Er, Yb: ZnO) nanopowders elaborated, using the sol-gel process would be a potential interesting material for UC applications.

## 1. Introduction

It has been established that the inability of Si photovoltaic cells to absorb low energy was one of the main shortcomings of these photovoltaic devices [1]. In this context, the up-conversion process is one of the proposed solutions to modify the incident solar radiation [2]. In up-conversion (UC), a high-energy photon is generated from the exploitation of energy by two or multiple low-energy incident photons [3]. In this context, the rare earth doped oxide type materials are good candidates for UC convertors in the back of the Si solar cell panel [4]. The low cost, non-toxicity, high thermal and chemical stability, high resistance, optical transparency, piezoelectric properties, and other properties of zinc oxide (ZnO) semiconductors make it a suitable host matrix for doping rare earth elements [5]. ZnO has a large band gap (3.37 eV) and is characterized by its high absorption and diffusion of ultraviolet radiation [6]. It is widely used in optoelectronics, catalysis, gas sensing, photoluminescence, and piezoelectric materials [7,8]. The structural and optical properties of ZnO can be controlled and enhanced by doping, due to its partially filled 4f energy level surrounded by full 5s and 5p orbitals. Doping by the Er/Yb pair, has received much interest in the literature and has been chosen for several photonic applications [9]. The Er^3+^ and Yb^3+^ ions present a spectral overlap between the ^2^F_5/2_→^2^F_7/2_ band of Yb^3+^ and the ^4^I_15/2_→^4^I_11/2_ band of Er^3+^, allowing the transfer of energy between these two rare earth ions, which enhances the UC process.

The synthesis routes of the doped ZnO nanoparticles are numerous, such as vapor deposition, precipitation in water solution, hydrothermal synthesis, precipitation from microemulsions, mechanochemical processes, and the sol-gel process [10]. Several studies have been conducted on the sol-gel synthesis of oxide material nanoparticles [11,12]. The sol-gel route has been considered as one of the most interesting techniques for the preparation of nanopowders, thanks to its versatility and its meticulously studied mass production potential [13]. This soft chemistry synthesis route is simple, low cost and makes it possible to have nanopowders with a size lower than 100 nm at low temperatures [14,15]. It also allows the control of the final properties of the elaborated powders [16]. Zinc acetate (Zn (CH3COO)_2_ .2H_2_O), Zinc chloride (ZnCl_2_), Zinc nitrate (Zn (NO_3_)_2_ .6H_2_O), and Zinc 2-ethylhexanoate (Zn (C_8_H_15_O_2_)_2_) are the primary precursors used in the synthesis of ZnO nanoparticles [17]. 

In the present work, undoped ZnO, (5 Er: ZnO), (5 Er, 5 Yb: ZnO), and (5 Er, 10 Yb: ZnO) were successfully prepared through the sol-gel process, using supercritical drying of ethyl alcohol. The Er/Yb doped ZnO nanopowders showed green and red emissions after excitation at 980 nm, via the UC mechanism. The energy band gap decreases after doping, and the electrical properties are improved. The effect of the Yb concentration on the crystallinity, UC emission, QY percentage, and electrical properties of the doped ZnO nanopowders was investigated in detail.

## 2. Materials and Methods

### 2.1. Samples Preparation

**Figure 1:**
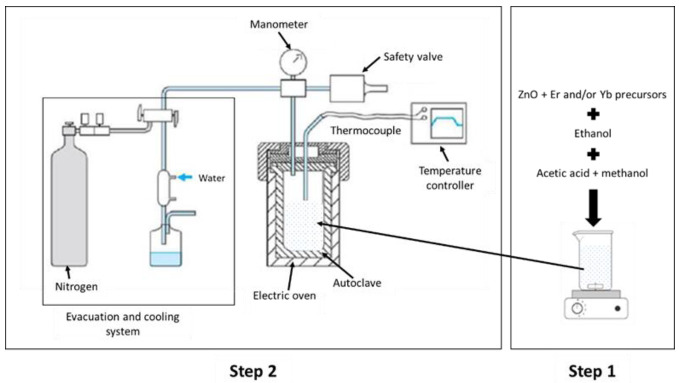
Preparation of the (Er, Yb: ZnO) nanopowders through the sol-gel method.

The development of the ZnO networks by the sol-gel process, took place via the inorganic polymerization reactions in the solution. These reactions were the hydrolysis at room temperature and condensation into an autoclave under high pressure until it reached the supercritical range of ethanol, in order to obtain an aerogel (Figure 1).

The solution of ZnO was prepared by dissolving a quantity of zinc acetate dihydrate (Zn(CH_3_COO)_2_.2H_2_O, >98%, Lobachemie), in ethanol (C_2_H_5_OH). For the (Er, Yb: ZnO) samples, 5% of Erbium (III) acetate hydrate (Er (CH_3_COO)_3_ XH_2_O, Strem Chemicals) and (5% or 10%) of Ytterbium (III) acetate hydrate (Yb (CH_3_COO)_3_ XH_2_O, Strem Chemicals) were added to the zinc precursor solution. The set was magnetically stirred at room temperature until the total dissolution of the precursors. Then, a mixture of acetic acid (C_2_H_4_O_2_) and methanol (CH_3_OH) was added to produce H_2_O by the esterification reaction and to ensure the hydrolysis of the solution in function of reaction (1):

Zn(OAc)_2_ + H_2_O → (OAc)–Zn + (Ac)–OH (1)

Next, the obtained mixture was introduced into an autoclave. The autoclave was closed to be heated and pressurized up to the supercritical range of ethanol (T = 241 °C, P = 63 bars) [18]. During this stage, a reaction of the condensation occurred in function of reaction (2):

(OAc)–Zn–OH + OH–Zn–(OAc) → (OAc)–Zn–O–Zn–(OAc) + H_2_O (2)

Following the drying cycle, a very slow expansion was performed up to the atmospheric pressure. Then, a nitrogen sweep at a low flow rate for 5 min was necessary to evacuate the rest of the solvent, remove the impurities, and reduce the mechanical stresses in the pores [19]. Finally, after 24 h, the autoclave was opened slowly to avoid cracking of the sample, due to thermal stresses and to obtain a thermal equilibrium. 

The recovered undoped ZnO, (5 Er: ZnO), (5 Er, 5 Yb: ZnO), and the (5 Er, 10 Yb: ZnO) nanopowders were annealed at 500 °C. The annealing was achieved in air at a heating rate of V = 2.5 °C/min and a holding time of t = 2 h. 

### 2.2. Measurements 

The crystallinity of the nanopowders was determined by XRD, using a Bruker D8 Advance X-Ray Diffractometer, equipped with a Cu Kα (λ = 1.54 Å) source (K780 X-ray generator operating at an applied voltage of 40 kV and an intensity of 40 mA). The detection set was made of a primary Germanium monochromator and a LynxEye 1D linear detector with 192 channels covering 3° in 2. An angle interval 2θ of 20° to 85° was recorded with a pitch of 0.05° using an integration time of 20 s.

The morphology and chemical compositions of the samples were examined by SEM imaging, using SEM-FEI Quanta FEG 250 with a hot cathode field effect for the electron emission. The accelerating voltage can reach 30 kV under a high vacuum (10-4 Pa). This device was equipped with an energy dispersive X-ray spectrometer (EDS). The morphology and particle size of the nanopowders (Er, Yb: ZnO) were examined through a transmission electron microscope (TEM), and the electron diffraction was performed on a JEOL 2011 microscope operating at 200 kV with a point-to-point resolution of 0.19 nm.

The purity of the ZnO nanopowders was checked by IR-FTIR spectroscopy with a Vertex 70v vacuum FTIR spectrometer, in a spectral range of 4000–250 cm^−1^ with a resolution of 4 cm^−1^. The nanopowders were spread out on the surface of Si wafers.

The UC spectra of the (Er, Yb: ZnO) samples were measured using a laser diode with a 980 nm excitation source and recorded in [500–750 nm] with a Jobin–Yvin U1000 spectrometer. An Edinburgh Instruments (FLS920) spectrofluorometer was used to measure the QY percentage of the nanopowders. To determine the dependence of the UC luminescence intensity upon the pumping power, the QY percentage and power density were measured by employing many filters with the same beam area for all of the measurements. The photoluminescence decays were recorded using a pulsed laser linked to a closed detection system.

The transmittance spectra were performed using a Lambda 950 spectrophotometer from Perkin Elmer in the range of 250–2500 nm.

The electrical properties were analyzed using 4-point probe resistivity measurements, using a PRO4 setup from Lucas Lab and a homemade Hall effect analyzer.

## 3. Results and Discussion

### 3.1. Structural and Morphological Studies

**Figure 2:**
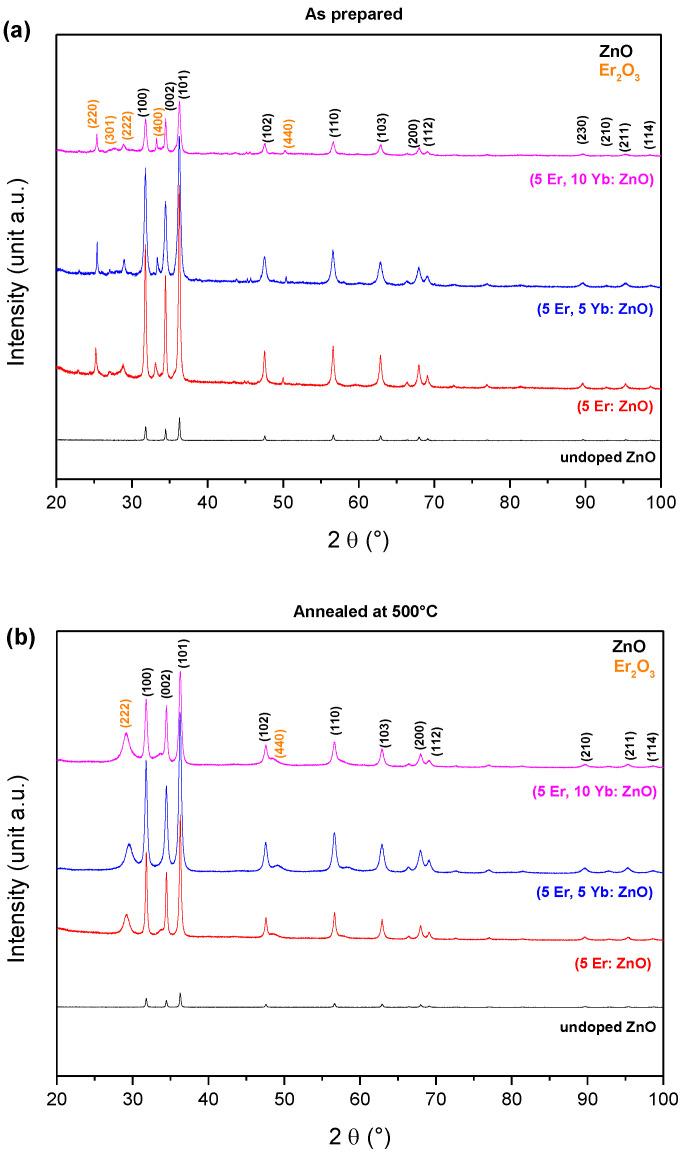
XRD patterns of the undoped ZnO, (5 Er: ZnO), (5 Er, 5 Yb: ZnO), (5 Er, 10 Yb: ZnO) nanopowders (**a**) as prepared and (**b**) annealed at 500 °C.

To study the effect of doping on the ZnO nanopowders, the XRD patterns of the undoped ZnO, (5 Er: ZnO), (5 Er, 5 Yb: ZnO), and (5 Er, 10 Yb: ZnO) aerogels, as prepared and annealed at 500 °C, are illustrated in Figure 2. All samples had a hexagonal structure of Wurtzite ZnO (space group P63mc, a = 3.249 Ӓ, c = 5.205 Ӓ, JPCDS 00-36-1451). The crystalline quality of the undoped ZnO aerogel was excellent at the prepared temperature (243 °C). In total agreement with Benammar et al. [20], no additional peak corresponding to a secondary phase or impurities was observed. This would confirm the purity of the powders developed through the sol–gel process. The appearance of new peaks assigned to the cubic structure of Erbium oxide Er_2_O_3_ and Ytterbium oxide Yb_2_O_3,_ after doping the ZnO with Er/Yb was observed. We totally agree with Hu et al.’s [21] and Yaoa et al.’s [22] interpretation, stating that because the atomic rays of Er^3+^ (0.89 Å) and Yb^3+^ (0.86 Å) are greater than those of Zn^2+^ (0.74 Å), it is difficult to incorporate all of the rare earth ions in the ZnO structures. Moreover, as was rightly reported by Chen [23] and Sangeetha, Muthukumaran and Ashokkumar [24], the formation of Er_2_O_3_ and Yb_2_O_3_ located at the grain boundaries would be caused by the coupling of the structure of the Er^3+^ and Yb^3+^ with the oxygen on the surface of the ZnO structure. 

**Figure 3:**
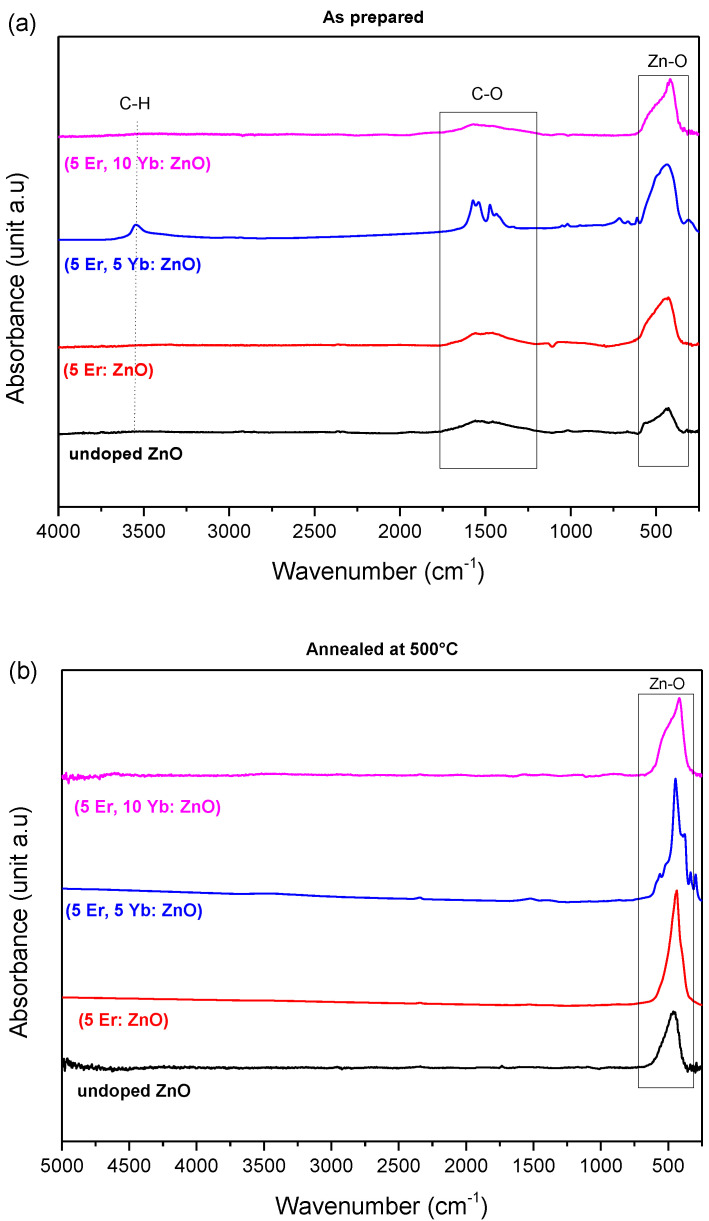
IR−FTIR spectra of the undoped ZnO, (5 Er: ZnO), (5 Er, 5 Yb: ZnO), (5 Er, 10 Yb: ZnO) nanopowders (**a**) as prepared and (**b**) annealed at 500 °C.

The Fourier transform infrared (FTIR) spectra of the undoped ZnO, (5 Er: ZnO), (5 Er, 5 Yb: ZnO), (5 Er, 10 Yb: ZnO) nanopowders, as prepared and annealed at 500 °C, are shown in Figure 3. The band between 413 and 510 cm^−1^ can be attributed to the vibration of the Zn-O bond. Azam et al. [25] rightly considered this band as a characteristic band of the hexagonal phase of Wurtzite ZnO. The FTIR spectrum of the as prepared nanopowders had bands at 1589 cm^−1^ and 2362 cm^−1^, attributed to the vibrations of the C-O bonds. Those carbonyl groups may be due to hydrocarbon contamination. The band located at 3550 cm^−1^ for the as prepared nanopowder may be attributed to the presence of alkyl groups (C-H) and would reflect an imperfection in the spectroscopic acquisition. Those bands would result from the experimental conditions that take place in the ambient air. Following the annealing at 500 °C, the bands of the carbonyl and alkyl groups disappeared, which would confirm the purity of the obtained nanopowders.

**Figure 4:**
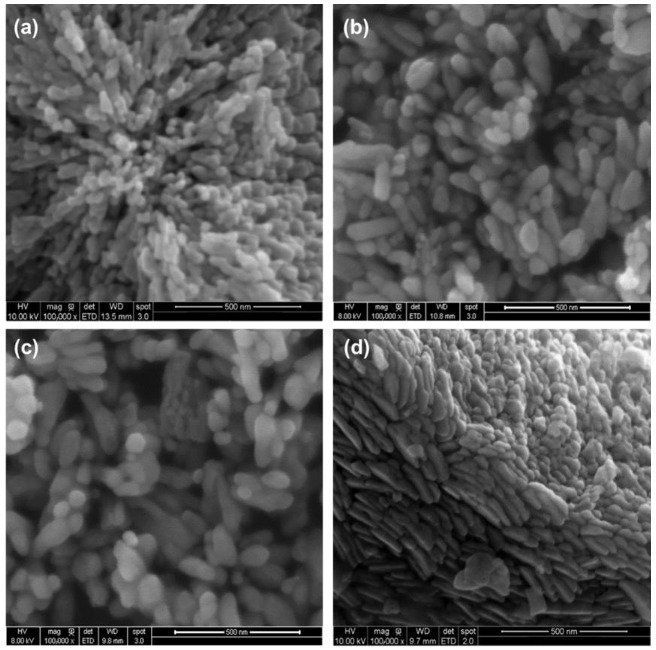
SEM images of (**a**) the undoped ZnO; (**b**) (5 Er: ZnO); (**c**) (5 Er, 5 Yb: ZnO); (**d**) (5 Er, 10 Yb: ZnO) annealed at 500 °C.

The SEM and TEM analyses were conducted to study the morphology and size of the nanopowders. Figure 4 shows the SEM images of the effect of the Yb doping concentration on the particle morphology of the (Er, Yb: ZnO) UC nanoparticles. It can be observed that the doping obviously changed the morphology of the nanoparticles. Since ZnO has a hexagonal structure, it has a C6v symmetry with the (0001) polar plane. This observation would confirms Zamiri et al.’s [26] finding that the (0001) basal plane has an important surface energy leading to the growth of the crystal along the C axis, which yields this spheroidal form of ZnO nanoparticles. The morphology obtained in this study was similar to that reported by Benhebal et al. [27] for the ZnO nanoparticles developed through the sol–gel process. The formation of a clear and highly packed grain structure was observed for 5 mol% doping of Yb, while the voids increased with an increase in the doping concentration and the size of the grains also changed with the doping concentration. 

**Figure 5:**
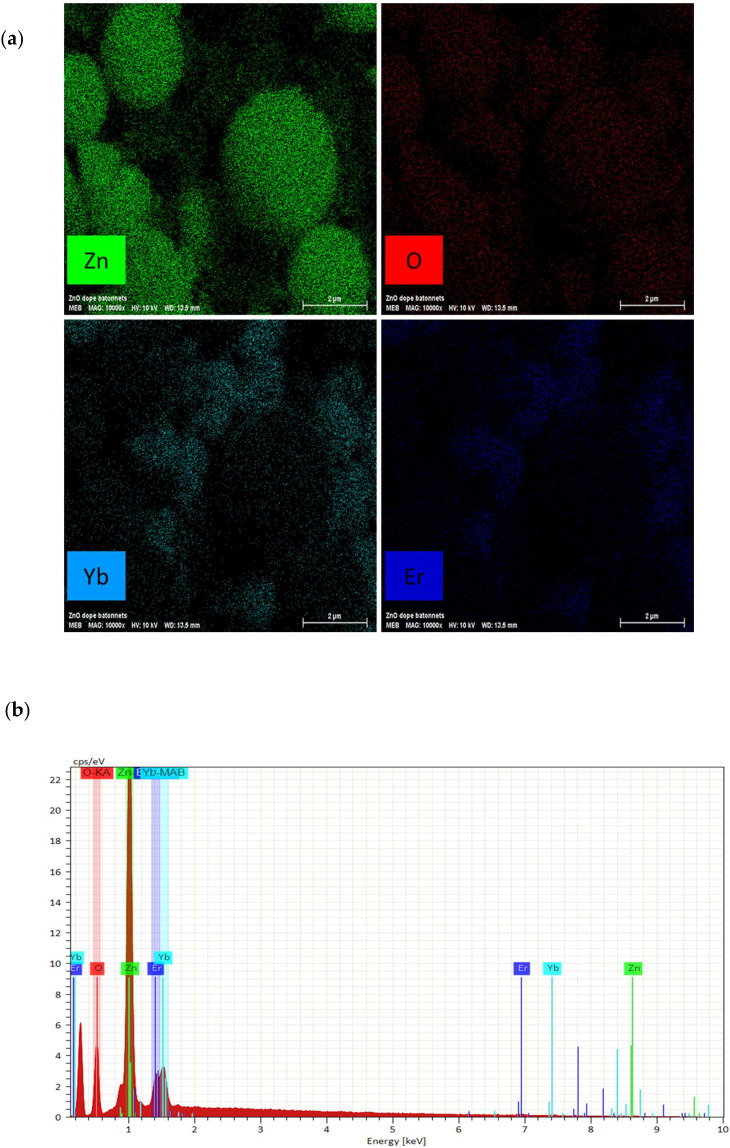
EDS (**a**) photos and (**b**) spectra of the (5 Er, 5 Yb: ZnO) nanopowder annealed at 500 °C.

Figure 5 shows the energy dispersive spectroscopy EDS (a) photos and (b) spectra of the (5 Er, 5 Yb: ZnO) nanopowder annealed at 500 °C. Both the EDS photos and spectra of this nanopowder confirmed the presence of Er and Yb, shown in dark blue and light blue, respectively, in the co-doped ZnO sample.

**Figure 6:**
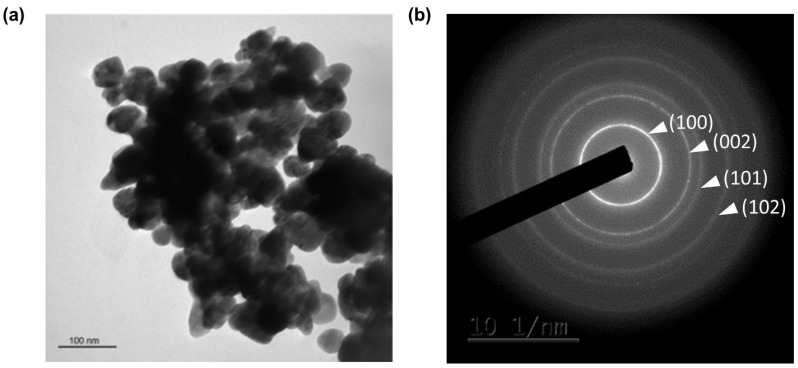
(**a**) TEM image and (**b**) selected area electron diffraction patterns (SAED) where diffraction planes corresponding to Wurtzite structure of (5 Er, 5 Yb: ZnO) nanopowder annealed at 500 °C.

Figure 6 illustrates the TEM images of (5 Er, 5 Yb: ZnO) annealed at 500 °C. The Figure 6a shows that the nanopowder appeared in the form of dense aggregates of crystallites. The average size of the crystallites is about 60 nm. The hexagonal structure of the doped ZnO nanopowder was confirmed by the selected area electron diffraction (SAED) technique in Figure 6b. The TEM observations corroborated with the XRD and SEM analyses, in terms of the grain structure and size measurements.

### 3.2. Up Conversion (UC) Emissions

**Figure 7:**
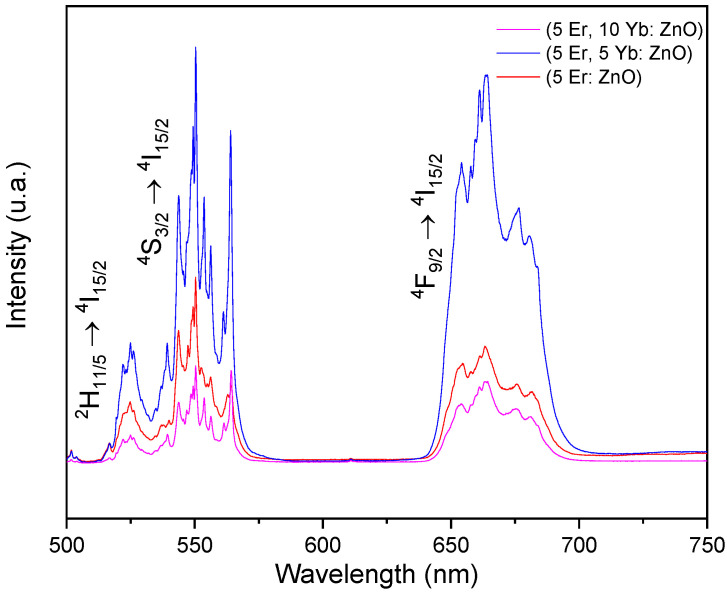
Room temperature UC emissions of the (5 Er: ZnO), (5 Er, 5 Yb: ZnO), (5 Er, 10 Yb: ZnO) nanopowder annealed at 500 °C, under 980 nm excitation.

Figure 7 shows the UC emission spectra of the (5 Er: ZnO), (5 Er, 5 Yb: ZnO) and (5 Er, 10 Yb: ZnO) nanopowders annealed at 500 °C after excitation at 980 nm at room temperature. There were two regions of visible emissions for all samples. Two emissions in the green region at 525 nm and 550 nm could be assigned to the ^2^H_11/5_ → ^4^I_15/2_ and ^4^S_3/2_ → ^4^I_15/2_ transitions, respectively, and an emission in the red region at 655 nm could be assigned to the red emission of the ^4^F_9/2_ → ^4^I_15/2_ transition. When compared to other samples, the (5 Er, 5 Yb: ZnO) annealed at 500 °C, had the highest UC emission intensity and the sharpest emission peaks. These findings correlate with Lim et al.’s [28] observations of other matrices doped by Er/Yb. The UC mechanism consists of absorbing two or more photons to produce energy good enough for the UC emission. The Yb ion is a sensitizer, which absorbs the incident energy and transfers it to the Er activator ion. The activator emits radiation, producing the luminescence of the UC particles.

**Figure 8:**
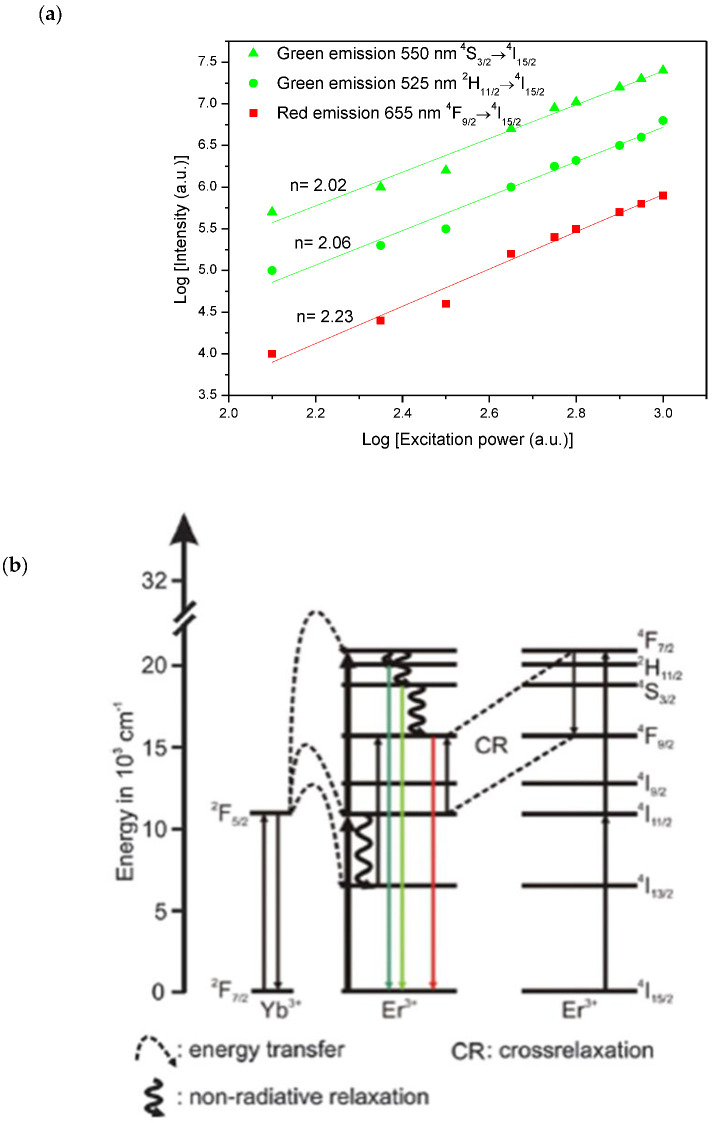
(**a**) Dependence of the UC luminescence intensity of (5 Er, 5 Yb: ZnO) upon the pumping power; and (**b**) energy level diagram of the Yb^3+^, Er^3+^ ions and the proposed UC mechanisms in (Er, Yb: ZnO) under 980 nm excitation.

Figure 8a illustrates the dependence of the UC luminescence intensity of (5 Er, 5 Yb: ZnO) upon the pumping power. The number of pumping photons n of the (5 Er, 5 Yb: ZnO) nanopowder was calculated from the slope of the intensity UC as a function of the laser excitation power. n = 2.02, 2.06, 2.23 were the values of 550 nm, 525 nm green emissions, and 655 nm red emissions, respectively. This finding would prove that the green and red emissions resulted from the two-photon UC process in this Er/Yb co-doped ZnO nanopowder.

To more clearly explain the results of the dependence of the UC luminescence intensity of (5 Er, 5 Yb: ZnO) upon the pumping power, the energy level diagram of the Yb^3+^ and Er^3+^ ions and the proposed UC mechanisms in (Er, Yb: ZnO) under 980 nm excitation, is illustrated in Figure 8b. The UC luminescence would be the origin of the combination of multiple processes, which are the ground state absorption (GSA), energy transfer (ET), excited state absorption (ESA), and cross relaxation (CR). The UC mechanism can be presented as follows:

(Yb^3+^) ^4^F_7/2_ + λ_980nm_ → ^4^F_5/2_

(Er^3+^) ^4^I_15/2_ + λ_980nm_ → ^4^I_11/2_

(Er^3+^) ^4^I_15/2_ + (Yb^3+^) ^4^F_5/2_ → (Er^3+^) ^4^I_11/2_ + (Yb^3+^) ^4^F_7/2_

(Er^3+^) ^4^I_11/2_ + (Yb^3+^) ^4^F_5/2_ → (Er^3+^) ^4^F_7/2_ + (Yb^3+^) ^4^F_7/2_

Because of its short lifetime, the fast and non-radiative relaxation of the level ^4^F_7/2_ would occur at the lower ^2^H_11/2_ and ^4^S_3/2_ levels. The green (525 nm and 550 nm) emissions would be, respectively, the results of the radiative transitions of ^2^H_11/2_ and ^4^S_3/2_ levels to the ground state level ^4^I_15/2,_ according to the following equations:

(Er^3+^) ^2^H_11/2_ → (Er^3+^) ^4^I_15/2_ + λ_525nm_

The red emission (655 nm) would be the result of a non-radiative relaxation from ^4^S_3/2_ to ^4^F_9/2_ level and a CR transition:

CR: (Er^3+^) ^4^F_7/2_ + (Er^3+^) ^4^I_11/2_ → (Er^3+^) ^4^F_9/2_

(Er^3+^) ^4^F_9/2_→ (Er^3+^) ^4^I_15/2_ + λ_660nm_

The difference in intensities of the ^4^S_3/2_ → ^4^I_15/2_ and ^2^H_11/2_ → ^4^I_15/2_ transitions can be justified by the rapid quenching of the second transition by non-radiative relaxation. The red emission intensity is lower than the green emission because of the low cross absorption relaxation.

**Figure 9:**
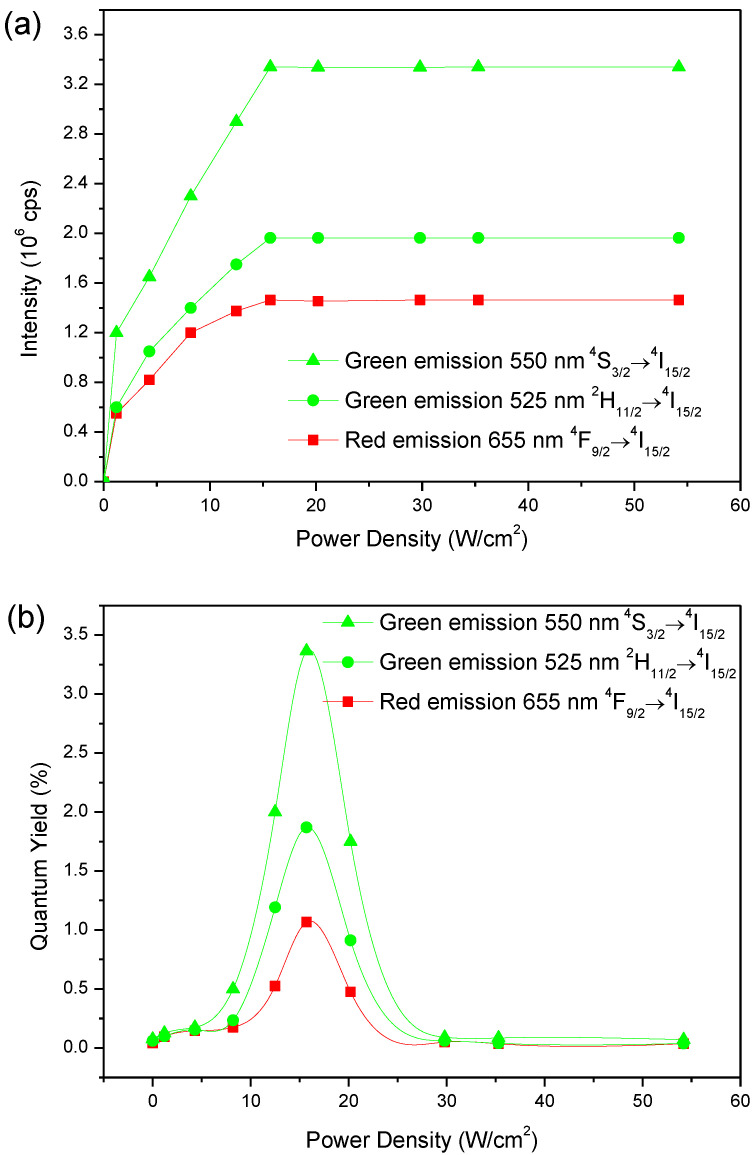
(**a**) UC intensity variations with an excitation power density for the (5 Er, 5 Yb: ZnO) nanopowder annealed at 500 °C with UC emissions centered at 525, 550 and 655 nm; (**b**) UC-QY percentage variation with excitation power density of (5 Er, 5 Yb: ZnO), annealed at 500 °C with UC emissions centered at 525, 550 and 655 nm.

In order to quantify the optical response of (5 Er, 5 Yb: ZnO) annealed at 500 °C, the percentages of UC-QY and the UC emission intensities centered at 525 nm, 550 nm, and 655 nm, were measured as a function of the power density under 980 nm excitation, respectively, in Figure 9a,b. We notice that the UC-QY percentage and the emission intensities depend on the power density. The emission intensity and the QY percentage increase until they reach a saturation threshold value of 15.7 W/cm^2^. This value was lower than the 20 W/cm2 of (3 Er, 17 Yb: ß-NaYF_4_) nanoparticles reported by Kaiser et al. [29]. Beyond this threshold saturation value (15.7 W/cm^2^), the QY percentages start to decrease, due to the saturation of the emitting states after excitation under 980 nm.

Table 1 shows a summary of the results exhibited in Figure 9b. These results reflect the QY percentages of the (Er, Yb, ZnO) nanopowders annealed at 500 °C at 15.7 W/cm^2^. The total QY percentage of (5 Er: ZnO), (5 Er, 5 Yb: ZnO), and (5 Er, 10 Yb: ZnO) was 4.59 ± 0.2%, 6.31 ± 0.2% and 3.77 ± 0.2%, respectively. As can be clearly seen in Figure 9b and Table 1, (5 Er, 5 Yb: ZnO) showed the highest values of absolute QY percentages in the green of 525 nm (1.87 ± 0.1%), in the green of 550 nm (3.37 ± 0.1%), and in the red (1.07 ± 0.2%) at 15.7 W/cm^2^, therefore the highest value of the total QY percentage (6.31 ± 0.2%). These results are very promising, when compared to those of (3 Er, 17 Yb: ß-NaYF_4_) with 0.32% at 20 W/cm^2^.

**Figure 10:**
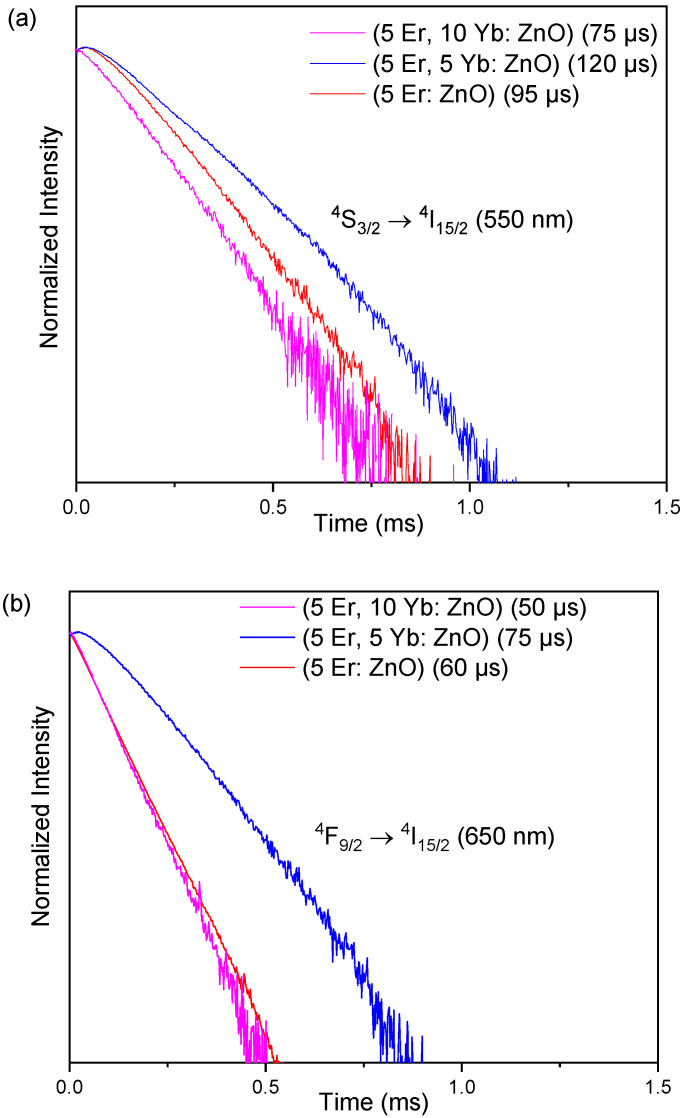
(**a**) The temporal evolution of the green (550 nm) ^4^S_3/2_ → ^4^I_15/2_ and (**b**) the temporal evolution of the red (655 nm) ^4^F_9/2_ → ^4^I_15/2_ emissions in the (Er, Yb: ZnO) nanopowders with different concentrations of Yb.

Figure 10a,b present the emission decay curves of (5 Er: ZnO), (5 Er, 5 Yb: ZnO) and (5 Er, 10 Yb: ZnO) at 550 nm (^4^S_3/2_ → ^4^I_15/2_) and 655 nm (^4^F_9/2_ → ^4^I_15/2_), respectively, under 980 nm excitation. As can be clearly seen, in Figure 10a, the longest excited state lifetime for the green emission had a value of 120 μs for (5 Er, 5 Yb: ZnO). Equally, Figure 10b shows that the longest excited state lifetime for the red emission, the decay time had a value of 75 μs for (5 Er, 5 Yb: ZnO). The relatively low decay time value for (5 Er, 10 Yb: ZnO) in both figures (75 μs in green and 50 μs in red), respectively, can be explained by the Er^3+^→Yb^3+^ energy back transfer rate. ^4^S_3/2_ (Er^3+^) + ^2^F_7/2_ (Yb^3+^) → ^4^I_15/2_ (Er^3+^) + ^2^F_5/2_ (Yb^3+^). This explanation correlates with Bergstrand et al.’s explanation of the phenomenon stating that the energy transfer depends on the distance between the Er and Yb ions as well as the dopant concentrations.

### 3.3. Optical Energy Band Gap

**Figure 11:**
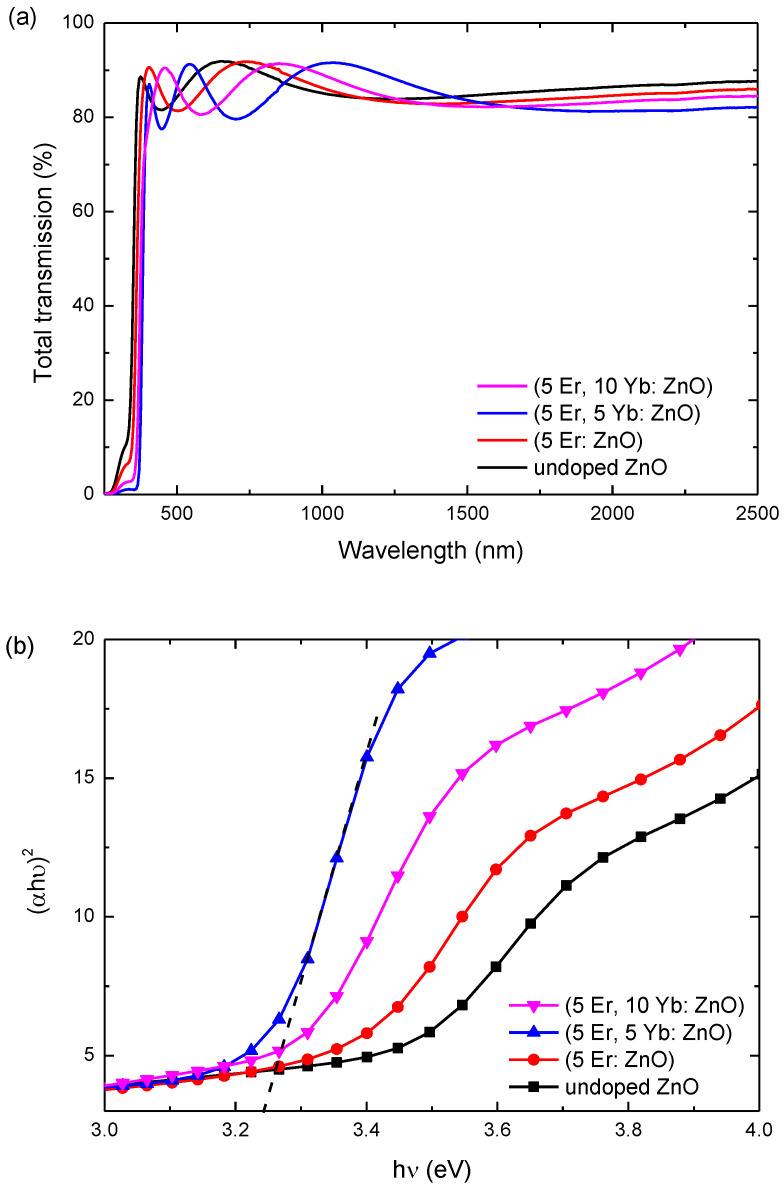
(**a**) Transmittance spectra of the undoped ZnO, (5 Er: ZnO), (5 Er, 5 Yb: ZnO) and (5 Er, 10 Yb: ZnO) annealed at 500 °C in the range of (0–2500) nm (**b**) the plots of the Kubelka–Munk function ((αhʋ)^2^) in the function of energy (hʋ).

Figure 11a exhibits the transmittance spectra of the undoped ZnO, (5 Er: ZnO), (5 Er, 5 Yb: ZnO) and (5 Er, 10 Yb: ZnO), annealed at 500 °C in the range of (0–2500) nm. For all samples, the transmittance values were greater than 86% in the range of 300 to 1500 nm. Moreover, all of the doped samples exhibited a transmittance value greater than that of the undoped ZnO. Figure 11b presents the plots of the Kubelka–Munk function ((αhʋ)^2^) in function of energy (hʋ), in order to calculate the energy gap of the undoped ZnO, (5 Er: ZnO), (5 Er, 5 Yb: ZnO) and (5 Er, 10 Yb: ZnO). Then, the optical energy band gap is calculated using the Tauc plot, according to the formula:

αhʋ = A(hʋ − E_g_)^n^

where α is the absorption coefficient, hʋ is the photon energy, A is constant, n = 1/2 is the power factor for the direct band gap crystalline semiconductors, and E_g_ is the energy band gap.

The optical band gap values of ZnO, (5 Er: ZnO), (5 Er, 5 Yb: ZnO) and (5 Er, 10 Yb: ZnO) are summarized in Table 2. As can be seen, after doping, the energy band gap decreased from 3.4 eV for the undoped ZnO to 3.34 eV, 3.24 eV and 3.3 eV for (5 Er: ZnO), (5 Er, 5 Yb: ZnO), and (5 Er, 10 Yb: ZnO), respectively. This can be explained by the formation of new bands of impurities and the strong interaction between the ZnO electrons and the electrons of rare earth ions. Indeed, as was rightly explained by Munawar et al [30] the doped rare earth ions form new intermediate energy bands in the ZnO lattice, below the conduction band, and play the role of the lowest empty molecular orbital, leading to the reduction of the band gap. In addition, the electron swapping between s-d and p-d orbitals causes a positive and a negative reorder between the valence band and the conduction band decreasing the band gap energy.

### 3.4. Electrical Properties

**Figure 12:**
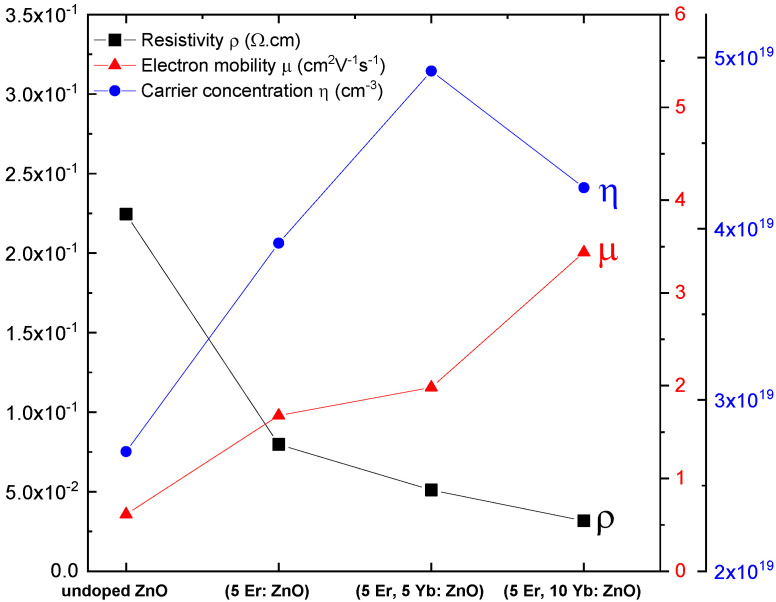
Resistivity (ρ), electron mobility (μ), and carrier concentration (η) with different concentrations of Yb.

The effect of the Yb concentration on the electrical properties of the (Er, Yb: ZnO) nanopowders was measured with the Hall effect. Figure 12 presents the resistivity (ρ), the electron mobility (μ), and the carrier concentration (η) with different concentrations of Yb. As can be seen, the resistivity decreased while the electron mobility increased after doping. Hence, it can be concluded that the doped nanopowders were n-type conductors. In line with Soumahoro et al. [31], this can be interpreted as an indicator of the presence of rarest earth atoms in interstitial positions, acting as donor impurities. The carrier concentration increased until doping (5 Er, 5 Yb: ZnO) then decreased to (5 Er, 10 Yb: ZnO). In total agreement with Asikuzun et al. [32] the increase in the carrier concentration can be explained by the substitution of the Zn^2+^ ions by the rare earth ions or by the incorporation of the rare earth ions in interstitial sites, while the decrease in the carrier concentration can be explained by the increase in the grain boundary defects that trap free carriers.

## 4. Conclusions

In conclusion, this study attempted to design and optimize a new up-converter from the (Er, Yb: ZnO) nanopowders using the sol–gel process to enhance the efficiency and reduce the cost of solar cells. The main findings of this study were: Firstly, the nanopowder (5 Er, 5 Yb: ZnO) would have the best emission intensity in the green at 525 nm and 550 nm and in the red at 655 nm. Secondly, this up-converter would have the best QY percentage with a value of 6.31 ± 0.2%, under a power density of 15.7 W/cm^2^. Thirdly, it had a lower band gap energy value of 3.24 eV and the best electrical properties. Fourthly, because of the availability of the materials and the relative easiness of the sol–gel process, the production of this nanopowder would have a lower cost than other materials. As a consequence, this optimized nanopowder would be recommended as a potential efficient up-converter and would be expected to enhance the efficiency of the solar cells and allow the harvest of more solar energy at a lower cost. Finally, this UC would be suitable for many other photonic applications. 

## Figures and Tables

**Table 1:** UC-QYs measured for the (Er, Yb: ZnO) nanopowders with different concentrations of Yb at their corresponding threshold excitation power densities.

QY Percentage at 15.7 W/cm^2^	Green (525 nm) (%)	Green (550 nm) (%)	Red (655 nm) (%)	Total QY Percentage
(5 Er: ZnO)	1.67 ± 0.2	2.07 ± 0.1	0.85 ± 0.1	4.59 ± 0.2
(5 Er, 5 Yb: ZnO)	1.87 ± 0.1	3.37 ± 0.1	1.07 ± 0.2	6.31 ± 0.2
(5 Er, 10 Yb: ZnO)	1.25 ± 0.2	1.85 ± 0.2	0.65 ± 0.2	3.77 ± 0.2

**Table 2:** Optical band gap calculated from the transmittance curve, using the Tauc formula.

Nanopowders	Optical Band Gap (eV)
Undoped ZnO	3.4
(5 Er: ZnO)	3.34
(5 Er, 5 Yb: ZnO)	3.24
(5 Er, 10 Yb: ZnO)	3.3

## Data Availability

Not applicable.

## References

[1] Schulze T.F., Czolk J., Cheng Y.-Y., Fückel B., MacQueen R.W., Khoury T., Crossley M.J., Stannowski B., Lips K., Lemmer U. (2012). Efficiency Enhancement of Organic and Thin-Film Silicon Solar Cells with Photochemical Upconversion. J. Phys. Chem. C.

[2] Nattestad A., Cheng Y.Y., MacQueen R.W., Schulze T.F., Thompson F.W., Mozer A.J., Fückel B., Khoury T., Crossley M.J., Lips K. (2013). Dye-Sensitized Solar Cell with Integrated Triplet–Triplet Annihilation Upconversion System. J. Phys. Chem. Lett..

[3] Cheng Y.Y., Fückel B., MacQueen R.W., Khoury T., Clady R.G.C.R., Schulze T.F., Ekins-Daukes N.J., Crossley M.J., Stannowski B., Lips K. (2012). Improving the light-harvesting of amorphous silicon solar cells with photochemical upconversion. Energy Environ. Sci..

[4] Chiappini A., Guddala S., Armellini C., Berneschi S., Cacciari I., Duverger-Arfuso C., Ferrari M., Righini G.C. Fabrication and Characterization of Colloidal Crystals Infiltrated with Metallic nanoparticles. Proceedings of the SPIE Proceedings [SPIE OPTO—San Francisco, California] Optical Components and Materials VII.

[5] Shukla S., Sharma D.K. (2020). A review on rare earths (Ce and Er)-doped zinc oxide nanostructures. Mater. Today Proc..

[6] Klingshirn C. (2007). ZnO: From basics towards applications. Phys. Status Solidi.

[7] Ali A., Phull A.-R., Zia M. (2018). Elemental zinc to zinc nanoparticles: Is ZnO NPs crucial for life? Synthesis, toxicological, and environmental concerns. Nanotechnol. Rev..

[8] Ozgür Ü., Alivov Y.I., Liu C., Teke A., Reshchikov M.A., Doğan S., Avrutin V., Cho S.-J., Morkoç H. (2005). A comprehensive review of ZnO materials and devices. J. Appl. Phys..

[9] Vijayalakshmi L., Kumar K.N., Hwang P., Kaur G. (2017). Enhancement of up-conversion emission and emerging cool white light emission in co-doped Yb^3+^/Er^3+^: Li_2_O-LiF-B_2_O_3_-ZnO glasses for photonic applications. Ceram. Int..

[10] Kołodziejczak-Radzimska A., Jesionowski T. (2014). Zinc Oxide—From Synthesis to Application: A Review. Materials.

[11] Hafdallah A., Derrar K., Aida M.S., Attaf N. (2016). Effet de la solution précurseur sur les propriétés structurales et otiques des couches minces de ZnO préparées par spray pyrolyse. Afr. Sci..

[12] Chen D., Huang F., Cheng Y.-B., Caruso R.A. (2009). Mesoporous Anatase TiO_2_Beads with High Surface Areas and Controllable Pore Sizes: A Superior Candidate for High-Performance Dye-Sensitized Solar Cells. Adv. Mater..

[13] Najafi A., Golestani-Fard F., Rezaie H., Saeb S.P. (2020). Sol-Gel synthesis and characterization of SiC–B4C nano powder. Ceram. Int..

[14] Al Abdullah K., Awad S., Zaraket J., Salame C.-T. (2017). Synthesis of ZnO Nanopowders by Using Sol-Gel and Studying Their Structural and Electrical Properties at Different Temperature. Energy Procedia.

[15] Najafi A., Sharifi F., Mesgari-Abbasi S., Khalaj G. (2022). Influence of pH and temperature parameters on the sol-gel synthesis process of meso porous ZrC nanopowder. Ceram. Int..

[16] Messaoud M., Trabelsi F., Kumari P., Merenda A., Dumée L.F. (2020). Recrystallization and coalescence kinetics of TiO_2_ and ZnO nano-catalysts towards enhanced photocatalytic activity and colloidal stability within slurry reactors. Mater. Chem. Phys..

[17] Hu X., Li G., Yu J. (2010). Design, Fabrication, and Modification of Nanostructured Semiconductor Materials for Environmental and Energy Applications. Langmuir.

[18] Faúndez C.A., Valderrama J.O. (2009). Activity Coefficient Models to Describe Vapor-Liquid Equilibrium in Ternary Hydro-Alcoholic Solutions. Chin. J. Chem. Eng..

[19] Sanusi Y.S., Mokheimer E.M. (2017). A numerical investigation of hydrogen production in an integrated membrane reformer-combustor. Energy Procedia.

[20] Benammar I., Salhi R., Deschanvres J.-L., Maalej R. (2017). The Effect of Solvents and Rare-Earth Element (Er, Yb) Doping on Suspension Stability of Sol-Gel Titania Nanoparticles. IEEE Trans. NanoBioscience.

[21] Hu J.Q., Li Q., Wong N.B., Lee C.S., Lee S.T. (2002). Synthesis of Uniform Hexagonal Prismatic ZnO Whiskers. Chem. Mater..

[22] Yao H., Shen H., Tang Q., Feng C., Li Y. (2019). Effect of Li co-doping with Er on up-conversion luminescence property and its temperature dependence of NaY(WO_4_)_2_. J. Phys. Chem. Solids.

[23] Chen W., Bovin J.-O., Joly A.G., Wang S., Su F., Li G. (2004). Full-Color Emission from In_2_S_3_ and In_2_S_3_:Eu^3+^ Nanoparticles. J. Phys. Chem. B.

[24] Sangeetha R., Muthukumaran S., Ashokkumar M. (2015). Structural, optical, dielectric and antibacterial studies of Mn doped Zn_0.96_Cu_0.04_O nanoparticles. Spectrochim. Acta Part A Mol. Biomol. Spectrosc..

[25] Azam A., Ahmed F., Arshi N., Chaman M., Naqvi A. (2010). Formation and characterization of ZnO nanopowder synthesized by sol-gel method. J. Alloys Compd..

[26] Zamiri R., Lemos A., Reblo A., Ahangar H.A., Ferreira J. (2013). Effects of rare-earth (Er, La and Yb) doping on morphology and structure properties of ZnO nanostructures prepared by wet chemical method. Ceram. Int..

[27] Benhebal H., Chaib M., Leonard A., Lambert S.D., Crine M. (2013). Photodegradation of phenol and benzoic acid by sol-gel-synthesized alkali metal-doped ZnO. Mater. Sci. Semicond. Process..

[28] Lim C.S., Aleksandrovsky A.S., Molokeev M.S., Oreshonkov A.S., Ikonnikov D.A., Atuchin V.V. (2016). Triple molybdate scheelite-type upconversion phosphor NaCaLa(MoO_4_)_3_:Er^3+^/Yb^3+^: Structural and spectroscopic properties. Dalton Trans..

[29] Kaiser M., Würth C., Kraft M., Hyppänen I., Soukka T., Resch-Genger U. (2017). Power-dependent upconversion quantum yield of NaYF_4_:Yb^3+^,Er^3+^ nano- and micrometer-sized particles—Measurements and simulations. Nanoscale.

[30] Munawar T., Nadeem M.S., Mukhtar F., Hasan M., Mahmood K., Arshad M., Hussain A., Ali A., Saif M.S., Iqbal F. (2021). Rare earth metal co-doped Zn0·9La0.05M0.05O (M = Yb, Sm, Nd) nanocrystals; energy gap tailoring, structural, photocatalytic and antibacterial studies. Mater. Sci. Semicond. Process..

[31] Soumahoro I., Schmerber G., Douayar A., Colis S., Abd-Lefdil M., Hassanain N., Berrada A., Muller D., Slaoui A., Rinnert H. (2011). Structural, optical, and electrical properties of Yb-doped ZnO thin films prepared by spray pyrolysis method. J. Appl. Phys..

[32] Asikuzun E., Ozturk O., Arda L., Terzioglu C. (2017). Microstructural and electrical characterizations of transparent Er-doped ZnO nano thin films prepared by sol-gel process. J. Mater. Sci. Mater. Electron..

